# Two-dimensional inverse problem of fire location in the closed goaf of coal mine based on optical fiber sensors

**DOI:** 10.1371/journal.pone.0298329

**Published:** 2024-02-27

**Authors:** Yujiao Liu, Lu Chen, Kaiping Wang, Zeyi Liu, Yao Zhang, Lianzeng Shi, Ke Gao, Zemiao Yang

**Affiliations:** 1 College of Safety Science and Engineering, Liaoning Technical University, Huludao, Liaoning, China; 2 Key Laboratory of Mine Thermo-Motive Disaster and Prevention, Ministry of Education, Huludao, Liaoning, China; Faculty of Engineering, University of Rijeka, CROATIA

## Abstract

Monitoring the temperature to determine the fire source locations is essential for controlling the spontaneous combustion in the goaf. Optical fiber sensors are employed to measure the temperature distribution in the goaf. However, due to changes in the geological conditions and the influence of the falling rocks in the goaf, only sensors on the upper side of the uncompacted goaf, due to inclination and coal pillar, may remain. Unilateral sensors are located on the upper side of the goaf, while fire occurs in the center. To investigate the issue with linear unilateral sensors, a two-dimensional inverse method has been developed to determine the location of fire sources by considering heat transfer after a fire inside the goaf. The equations were theoretically solved using Green’s function method to obtain the internal temperature distribution of the physical model of the goaf. Sensitivity analysis identified the most crucial parameters in the process of spontaneous heating at different temperature. The fire source location can be determined using a loop method based on the model calculations. We considered a case to validate the model. Accurately identifying the fire source location in the goaf using the unilateral sensors has an essential theoretical and practical significance for fire prevention and fighting.

## Introduction

The development of the mining industry poses a serious threat to miners, mine equipment and resources due to fires in underground mines. Fires resulting from spontaneous combustion of residual coal in coal mine goafs have been a significant hazard threatening the safety of miners [1,2]. The oxidation reaction of the residual coal with leaked air in the goaf leads to a temperature rise, a cricial factor in identifying the fire source location and precenting spontaneous combustion in the goaf. Fires typically occur at goaf depths, but their location shifts during mining due to the spatial stress distribution of rock mass in the goaf [3], making accurate fire location determination challenging [4]. This hazard escalates if the fire location can’t be identified. Therefore, preventing and controlling coal seam fires require, as a primary step, accurately localizating spontaneous combustion of residual coal.

The fire source location provides information about changes in the physical and chemical properties of coal and its surrounding rocks. Technologies for the fire source location includes temperature [5–8], gas concentration [9–12], radon concentration [13–15], magnetic field [16], resistivity [17], and numerical simulation [18], et al. Zhang et al [19] used oxygen concertation as the indicator to identify the residual coal spontaneous combustion-prone region. Pi et al [20] laid thermocouple thermometry and bundle tube monitoring system into the goaf to measure the fire location. Lei et al [21] introduced random forest (RF) and support vector machine (SVM) to predict coal spontaneous combustion based on the in-situ monitoring data. Kong et al [22] got a positive correlation between the electromagnetic radiation signals and coal temperature and presented the conceptual design of a method to detect concealed fire. Zhang et al [23] integrated soot concentration and temperature data to compile a comprehensive dataset for the task of fire location detection in ultra-wide immersed tube tunnels.

Temperature is a crucial parameter closely associated with coal spontaneous combustion. Sensors can measure the variation of temperature field in the goaf during the mining process, and the possibility of fire in the goaf can be evaluated based on the temperature field [24]. Temperature data in the goaf can be acquired using the borehole temperature measurements [25]. A numerical simulation method was developed to quickly identify the high-temperature zone of the fire by simulating oxygen concentrations and temperature distributions in the goaf [26,27]. The GtmWSN model was proposed using a wireless sensor network, and a data transmission scheme was developed to monitor goaf temperature [28]. Liang et al [29] studied to determine a suitable heating range for infrared gas sensors and established an error optimization model to study an in-situ online monitoring system for coal mine gas. A distributed optical fiber temperature sensing system based on Raman scattering was presented and applied in the goaf in recent years [30]. The optical fiber detection technology for measuring temperature in the goaf has been increasingly emphasized.

Optical fiber sensors are currently employed to measure temperature and predict fire trend in the goaf. Howere, these sensors can be easily damaged by falling rocks [10]. Only sensors on the upper side of the goaf, not compacted due to inclination and coal pillar, might remain. The remaining sensors are on the upper side of the goaf, while the fire occurs in the center. Precise identification of the fire source location in the goaf by the remaining sensors holds crucial theoretical and practical importance for the fire prevention and fighting. To adress the problem with linear remaining sensors, this study proposes a new theoretical inverse method to detect the location of fire sources in the goaf. The heat transfer mathematical model in the mining area is simplified and constructed to analyze the complex flow environment in the goaf based on the heat transfer characteristic of the mining area. The Green’s function method is used to solve the analytical solution for the temperature in the extraction zone under the condition of a single-point fire source. The temperature model of the internal field of the extraction zone is derived, and the study also exploreds the effects of the fire source intensity, heat generation time, thermal conductivity and different fire source locations on the temperature field of the extraction zone.

## 2D inverse model for spontaneous combustion in the goaf

### Temperature field model in the goaf

Heat transport in the goaf of coal mine can result from conduction and convection [31,32]. The goaf of a fully mechanized coal mine undergoes two stages periodically: random accumulation and rock block recompression. Cracks and holes in rock blocks significantly impact the space of the fluid channels in the goaf where the fluid flow. The flow field and temperature field can be calculated by porous media fluid dynamics theory. Temperature transfer depends on lots of factors. Therefore, the following simplification and hypotheses are introduced.

- The goaf is regarded as a homogeneous and isotropic porous medium.
- The thermal conductivity of the goaf is treated as the comprehensive equivalent thermal conductivity.
- Conduction primarily happene through coal or rock particles. Conduction through air can be disregarded due to air’s relatively low thermal conductivity. Ignoring the heat radiation of the fire source. The thermal physical properties (thermal conductivity, specific heat) of coal and rock are regarded as constants, and their temperature-induced changes are not considered.
- The water phase in the goaf remain constant, and the effect of water migration on heat transfer is not considered.
- The temperature of the collapsing rocks in the goaf is not significantly different from the sur-rounding temperature. Thus it is assumed that the initial temperature inside the goaf is the same.
- The wind speed remains constant, and the convective heat transfer coefficient between the surrounding rock and the wind is constant.

**Fig 1:**
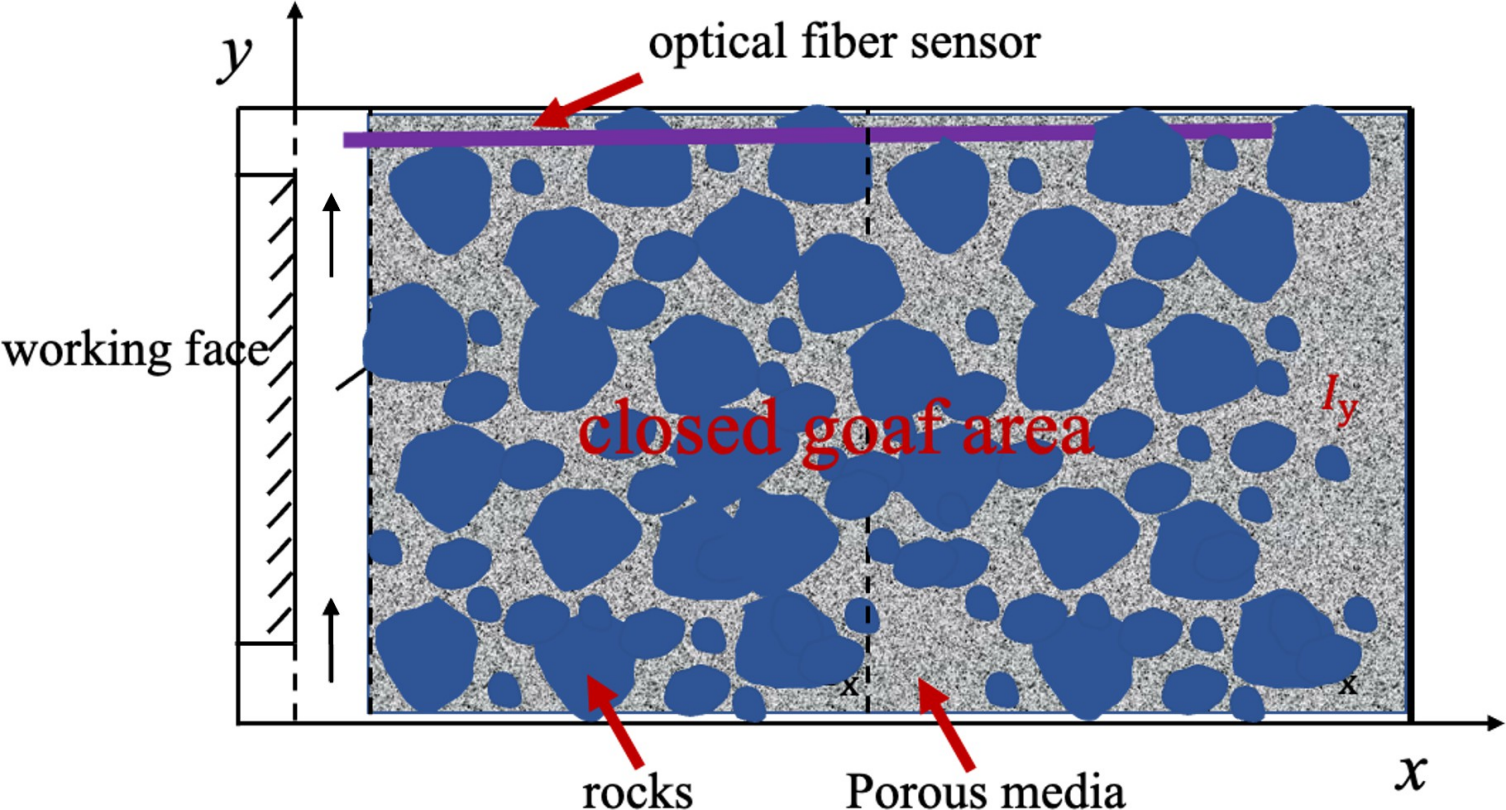
Physical model of the goaf.

In this paper, the goaf is represented by the simplified physical model shown in Fig 1. The ’*l*_y_’ shown in Fig 1 represents the longitudinal coordinates of the goaf model; ’ *x*’ represents the lateral position of the model; The arrow indicates the direction of the airflow.The mathematical model of the temperature field of the goaf is established according to the two-dimensional physical model of the depth and width of the goaf.

Based on the two-dimensional heat transfer mathematical model in the goaf, the analytical expression of the goaf temperature field is derived as:

$$\left\{ \begin{array}{l} \alpha (\frac{\partial^{2}\theta }{\partial x^{2}}+\frac{\partial^{2}\theta }{\partial y^{2}})+\frac{q}{\rho c}=\frac{\partial \theta }{\partial t},0<x<a,0<y<b,>0\\ \frac{\partial \theta }{\partial x}=0,x=0,-\lambda \frac{\partial \theta }{\partial x}=H_{1}\theta ,x=a,t>0\\ \frac{\partial \theta }{\partial y}=0,y=0,-\lambda \frac{\partial \theta }{\partial y}=H_{2}\theta ,y=b,t>0\\ \theta_{0}=T,0<x<a,0<y<b,t=0 \end{array}\right.$$ (1)

Where *α* is the thermal diffusivity inside the goaf, m^2^/s, *θ* is the temperature, °C, *q* is the fire intensity, W/m^3^, *ρ* is the density, kg/m^3^, *c* is the specific heat capacity of the goaf, kJ/(kg·°C), *t* is time, s, *λ* is the thermal conductivity of the goaf, W/(m·°C), *H*_1_ is the convective heat transfer coefficient between wind flow and the goaf boundary *x* = *a*, W/(m^2^°C), *H*_2_ is the convective heat transfer coefficient between wind flow and the goaf boundary *y* = *b*, W/(m^2^°C).

The instantaneous ignition source whose intensity is equal *ρc* to the quantity can be written q=ρcδ(x−x')δ(y−y')δ(t−t') at a point (*x*’,*y*’). The temperature distribution caused by such a fire source under homogeneous boundary conditions and initial conditions is Green’s function *G*. Green’s function *G* satisfies the following auxiliary problems.


$$\left\{ \begin{array}{l} \alpha (\frac{\partial^{2}G}{\partial x^{2}}+\frac{\partial^{2}G}{\partial y^{2}})+\delta (x-x^{\prime })\delta (y-y^{\prime })=\frac{\partial G}{\partial t},0<x<a,0<y<b,t>0\\ \frac{\partial G}{\partial x}=0,x=0,-\lambda \frac{\partial G}{\partial x}=H_{1}\theta ,x=a,t>0\\ \frac{\partial G}{\partial y}=0,y=0,-\lambda \frac{\partial G}{\partial y}=H_{2}G,y=b,t>0\\ G=0,0<x<a,0<y<b,t=0 \end{array}\right.$$ (2)

According to the law of conservation of energy, the heat absorbed by all positions (*x*’,*y*’) in the goaf from *t* = *t*’−0 to *t* = *t*’ is equal to the energy generated by the ignition source, that is, the effect of the instantaneous ignition source is equivalent to the initial temperature distribution at *t*’. The Eq (2) can be converted to the following ones.


$$\left\{ \begin{array}{l} \alpha (\frac{\partial^{2}G}{\partial x^{2}}+\frac{\partial^{2}G}{\partial y^{2}})=\frac{\partial G}{\partial t},0<x<a,0<y<b,t>0\\ \frac{\partial G}{\partial x}=0,x=0,-\lambda \frac{\partial G}{\partial x}=H_{1}\theta ,x=a,t>0\\ \frac{\partial G}{\partial y}=0,y=0,-\lambda \frac{\partial G}{\partial y}=H_{2}G,y=b,t>0\\ G=\delta (x-x^{\prime })\delta (y-y^{\prime }),0<x<a,0<y<b,t=0 \end{array}\right.$$ (3)

The Eq (3) can be solved using the separation of variables method.


$$G(x,y,t)=\sum_{\begin{array}{l} m=1\\ n=1 \end{array}}^{\infty }C_{mn}\cos(\beta_{m}x/a)\cos(\gamma_{n}y/b)\exp(-\alpha [{\left(\beta /a\right)}^{2}+{\left(\gamma /b\right)}^{2}]t)$$ (4)

Where, *β*_*m*_ is the transcendental equation $\tan(\beta_{m})=aH_{1}/\lambda \beta_{m}$, *γ*_*n*_ is the transcendental equation $\tan(\gamma_{m})=bH_{2}/\lambda \gamma_{m}$.

*C*_*mn*_ can be determined by G=δ(x−x')δ(y−y') at *t* = 0.


$$\delta (x-x^{\prime })\delta (y-y^{\prime })=\sum_{\begin{array}{l} m=1\\ n=1 \end{array}}^{\infty }C_{mn}\cos(\beta_{m}x/a)\cos(\gamma_{n}y/b)\exp(-\alpha [{\left(\beta /a\right)}^{2}+{\left(\gamma /b\right)}^{2}]t)$$ (5)

The orthogonal function series can be used to expand the function and *C*_*mn*_ can be obtained.


$$\begin{array}{c} C_{mn}=\sum_{\begin{array}{l} m=1\\ n=1 \end{array}}^{\infty }\frac{4\beta_{m}\gamma_{n}}{ab(\beta_{m}+\sin\beta_{m}\cos\beta_{m})(\gamma_{n}+\sin\gamma_{n}\cos\gamma_{n})}\cos(\beta_{m}x^{\prime }/a)\cos(\gamma_{n}y^{\prime }/b)\\ \exp{\left(-\alpha [(\beta_{m}/a\right)}^{2}+{\left(\gamma_{n}/b\right)}^{2}]t^{\prime }) \end{array}$$ (6)

Bring Eq (6) into Eq (4).


$$\begin{array}{c} G=\sum_{\begin{array}{l} m=1\\ n=1 \end{array}}^{\infty }\frac{4\beta_{m}\gamma_{n}}{ab(\beta_{m}+\sin\beta_{m}\cos\beta_{m})(\gamma_{n}+\sin\gamma_{n}\cos\gamma_{n})}\cos(\beta_{m}x^{\prime }/a)\cos(\gamma_{n}y^{\prime }/b)\cos(\beta_{m}x/a)\cos(\gamma_{n}y/b)\\ \exp{\left(-\alpha [(\beta_{m}/a\right)}^{2}+{\left(\gamma_{n}/b\right)}^{2}](t-t^{\prime })) \end{array}$$ (7)

The analytical solution function of the temperature field in the goaf can be obtained from Green’s function *G*.


$$\begin{array}{l} \theta (x,y,t)=\sum_{\begin{array}{l} m=1\\ n=1 \end{array}}^{\infty }\frac{4\theta_{0}\sin\beta_{m}\sin\gamma_{n}}{\left(\beta_{m}+\sin\beta_{m}\cos\beta_{m})(\gamma_{n}+\sin\gamma_{n}\cos\gamma_{n}\right)}\cos(\beta_{m}x/a)\cos(\gamma_{n}y/b)\exp{\left(-\alpha [(\beta_{m}/a\right)}^{2}+{\left(\gamma_{n}/b\right)}^{2}]t)\\ \;+\sum_{\begin{array}{l} m=1\\ n=1 \end{array}}^{\infty }\frac{4ab\beta_{m}\gamma_{n}q_{p}}{\alpha (\beta_{m}+\sin\beta_{m}\cos\beta_{m})(\gamma_{n}+\sin\gamma_{n}\cos\gamma_{n})(b^{2}\beta^{2}+a^{2}\gamma^{2})}\cos(\beta_{m}x^{\prime }/a)\cos(\gamma_{n}y^{\prime }/b)\cos(\beta_{m}x/a)\\ \;•\cos(\gamma_{n}y/b)[1-\exp{\left(-\alpha [(\beta /a\right)}^{2}+{\left(\gamma /b\right)}^{2}]t)] \end{array}$$ (8)

### Inverse model based on temperature field and test data

**Fig 2:**
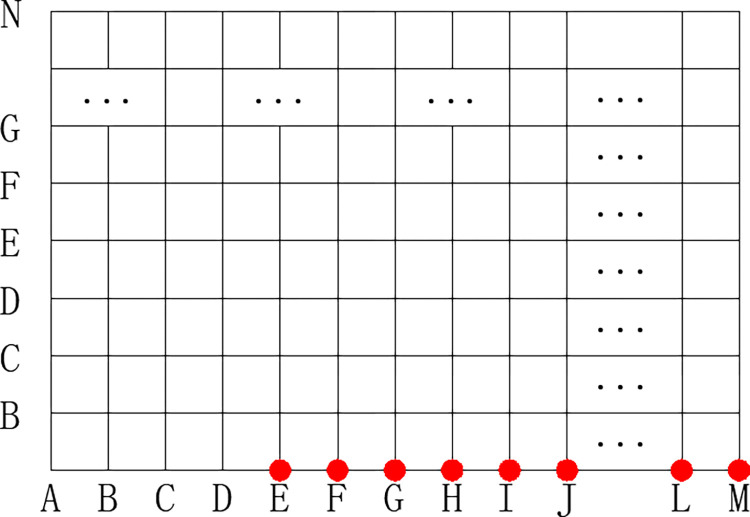
Divided grids of the goaf area.

The goaf area Ω = {0<x<a,0<y<b} is divided into the grids *m*×*n* in Fig 2. the nodes *EA*, *FA*,…,*MA* are selected as the temperature measurement point, and their coordinate position corresponds to the actual measurement position of the optical fiber. The inversion process is as follows. (1) Consider the *m*×*n* nodes on the grid as individual heat source points and input the node coordinates into the temperature field analytic function to derive different temperature analytical values for the same point under varying heat source coordinates. (2) Take each node as a heat source point and input it into the gob temperature field analytic function to obtain the temperature values for nodes *EA*, *FA*,…,*MA* under the conditions of different heat source point coordinates. (3) Determining the fire source area by minimizing the difference between the analytical value and the measured value of the temperature measurement point using the least-squares model. According to the least squares model, the function *E* representing the difference between the analytical value and the measured value of the temperature measurement point is established. The location of the fire source is where *E* is the minimized at (*x*_0_, *y*_0_), (*x*_0_, *y*_0_) is the location of the fire source.


$$E=\sum_{i=1}^{i=N}\sqrt{{\left(\theta_{mn}{\left(x,y,t\right)}_{i}-T{\left(x,y,t\right)}_{i}\right)}^{2}}$$ (9)

Where, *E* is a deviation between the analytical values *θ*(*x*, *y*)_*i*_ and the measured value *T*(*x*, *y*)_*i*_, *N* is the number of temperature measuring points, *mn* are the coordinates of the fire source for calculating the temperature.

### Analysis of the effect of different thermal physical parameters on the temperature field in the mining area

To investigate how various thermal properties impact temperature field within the extraction zone, we utilize the theoretical model diagram for temperature field analysis. The internal thermal properties parameters include a thermal conductivity of 1.8 W/(m·°C), a density of 1100 kg/m^3^, a specific heat capacity of 830 kJ/(kg·°C), and an initial internal temperature of 20°C. The model dimensions is chosen to be a = 1m, b = 1m, the interior is filled with rock particles, and the convective heat transfer coefficient and the engineering simplified formula of the analytical solution of heat transfer in the extraction zone proposed in this chapter are applied to calculate the temperature field in the extraction zone.

Due to the small thermal diffusion coefficient and thermal conductivity of the rocks, the temperature variation is not significant when applying the analytical solution to a large quarry area. To examine the impact of various factors on the temperature field more clearly, this paper opts to restrict the calculation range of the quarry area to 1m*1m, facilitating easier comparision of temperature variation images. To observe the change of temperature gradient in the extraction area and the subsequent can make a concise and effective analysis of the calculation results, the coordinate points (0.4, 0.4), (0.6, 0.6), (0.7, 0.7), (0.8, 0.8), (0.9, 0.9) five points are selected as the observation points (as shown in Table 1), so that it is easy to compare and analyze the changes of different influencing factors on the temperature values in the system. This section focuses on the effects of heat source intensity, heat generation time, thermal conductivity, and the location coordinates of different points on the temperature field within the extraction zone; therefore, the thermal diffusion coefficient and convective heat transfer coefficient are set as constant values.

**Table 1:** Location of temperature measuring point.

Temperature measurement point location number	Location Coordinates /m	
	CH01 x	y
CH01	0.4	0.4
CH02	0.6	0.6
CH03	0.7	0.7
CH04	0.8	0.8
CH05	0.9	0.9

#### The effect of heat source intensity on the temperature field

To investigate the impact of different heat source intensities on the temperature field, we consider the heat source intensity calculation conditions outlined in Table 2. These conditions are based on the heat intensity of the coal body in the mining area, ranging from the self-heating stage to the combustion stage.

**Table 2:** Calculation conditions of different heat source intensity.

Number	Heat source intensity	Heat generation time	Thermal conductivity	Location Coordinates
	W/m^2^	s	W/(m^2^⋅°C)	m
1	100	10800	1.8	CH02
2	200	10800	1.8	CH02
3	300	10800	1.8	CH02
4	400	10800	1.8	CH02
5	500	10800	1.8	CH02
6	600	10800	1.8	CH02
7	700	10800	1.8	CH02
8	800	10800	1.8	CH02
9	900	10800	1.8	CH02
10	1000	10800	1.8	CH02

**Fig 3:**
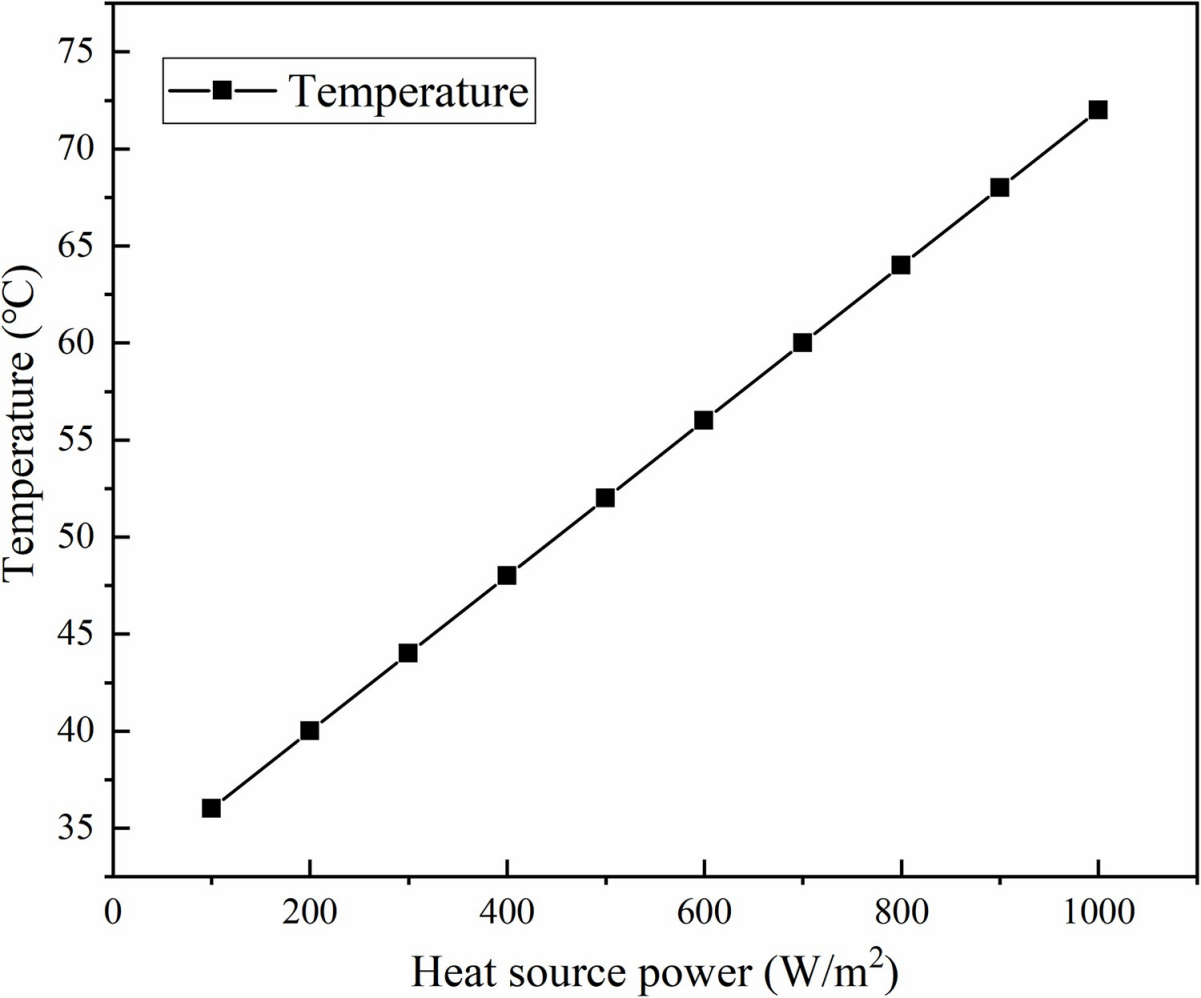
Influence of different heat source intensity on temperature.

The effect of different heat source intensities on the temperature values after 3h of heat source heating time at observation point CH01 is shown in Fig 3. As can be seen from the figure, when the heat source intensity is 100, the temperature value on observation point CH01 is 45.84℃, and with the increase of heat source intensity, when the heat source intensity is 1000, the temperature value on observation point CH01 is 81.2℃, it can be seen that the difference of temperature values of observation point temperature observation point is very obvious, and the relationship is positive, that is, with the increase of heat source intensity, the monitoring point temperature value also increases with the increase of heat source intensity.

#### Effect of thermal conductivity on temperature field

To study the effect of different thermal conductivity on the temperature field of the heat source and to analyze the range of thermal conductivity of coal rocks in different mining areas, the main calculation parameters conditions were selected as shown in Table 3.

**Table 3:** Calculation conditions of different thermal conductivity.

Number	Heat source intensity	Heat generation time	Thermal conductivity	Location Coordinates
	W/m^2^	s	W/(m^2^⋅°C)	m
1	800	0–10800	1.8	CH02
2	800	0–10800	2.0	CH02
3	800	0–10800	2.2	CH02
4	800	0–10800	2.4	CH02

**Fig 4:**
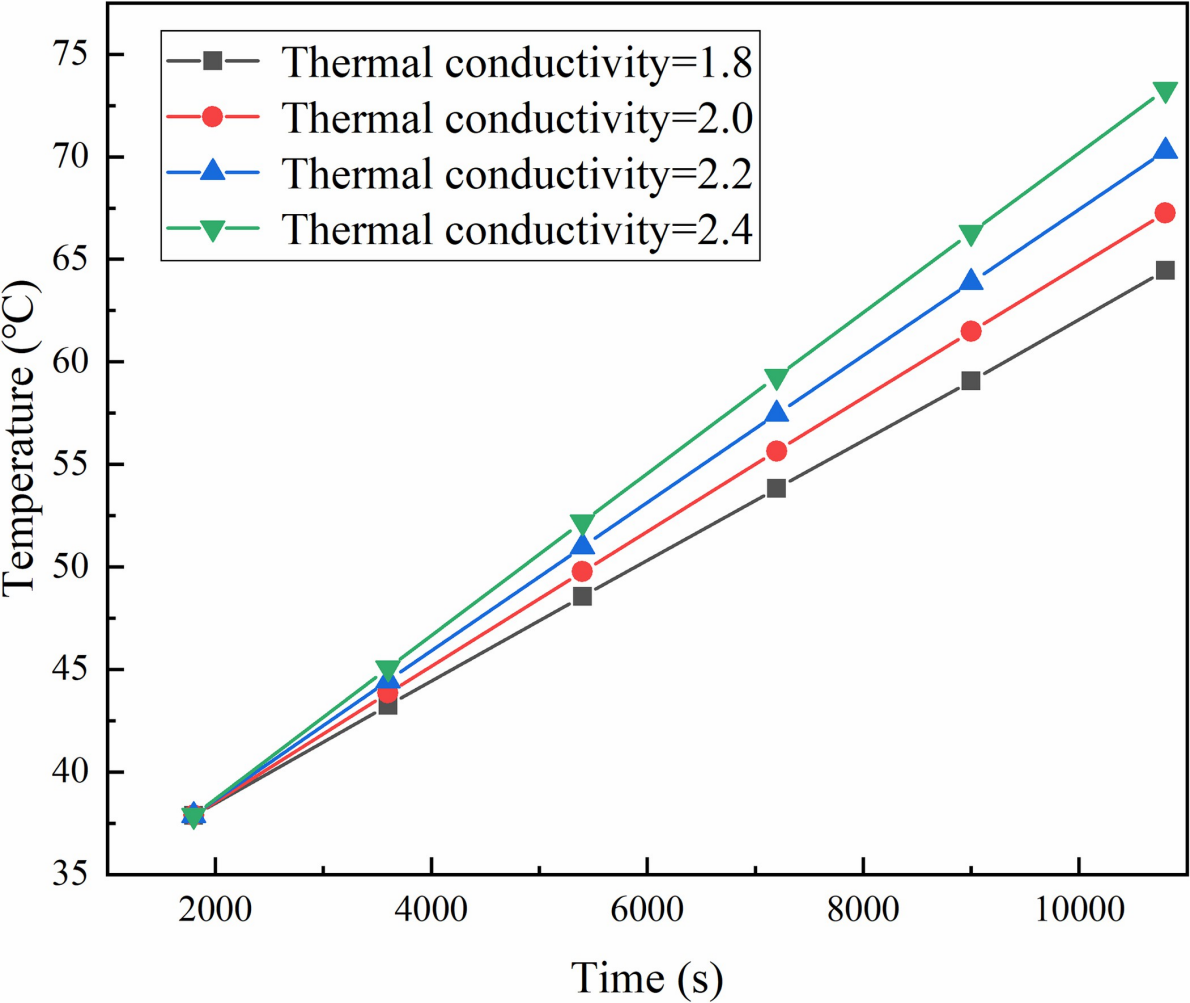
Influence of different thermal conductivity on temperature.

The temperature changes at various observation points were calculated after 3h with different thermal conductivity, as illustrated in Fig 4. It is observed that as thermal conductivity increases, the temperature rises at all measurement points, However, the higher the thermal conductivity, the faster the rate of temperature increase. For various observation points, proximity to the heat source results in higher temperature and faster the rate of temperature increase. Similarly, for a given observation point, higher selected thermal conductivity leads to increased temperature values and faster rate of temperature increase. Additionally, at 1800s heating time, temperature differences among the observation points are not significant, indicating that the effect of different thermal conductivity on the temperature is not apparent. However, with tprolonged heating time, higher thermal conductivity corresponds to higher temperatures and a greater rate of temperature increase.

#### Effect of heating time on temperature field

In order to analyze the effect of increasing heating time on the temperature values at the observation point CH01, when the other thermal physical parameters are fixed values. The conditions of the calculated parameters were chosen as shown in Table 4.

**Table 4:** Calculation conditions of different heating time.

Number	Heat source intensity	Heat generation time	Thermal conductivity	Location Coordinates
	W/m^2^	s	W/(m^2^⋅°C)	m
1	800	10800	1.8	CH02

**Fig 5:**
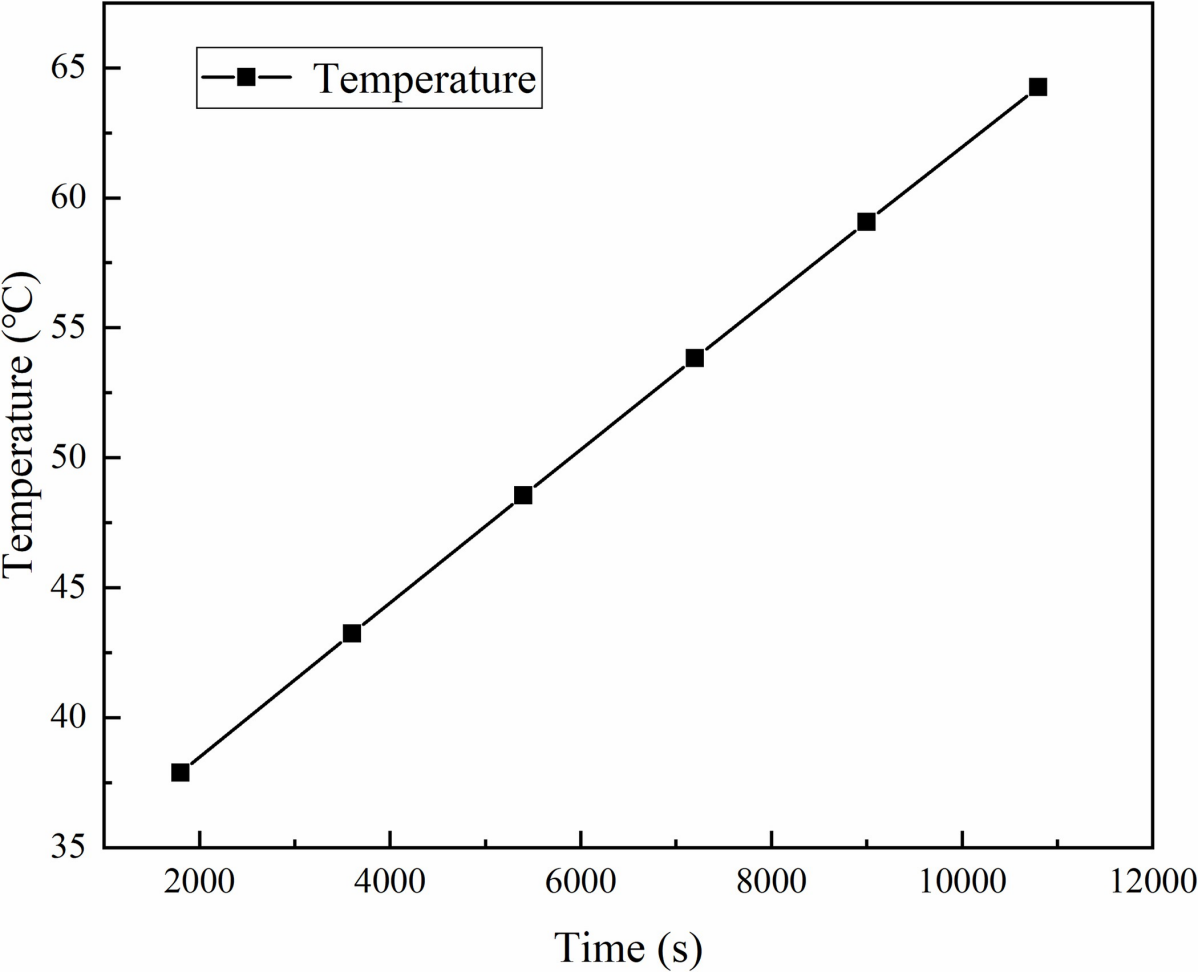
Influence of different time on temperature.

Fig 5 shows that at 1800s, the temperature at observation point CH01 is 43.5℃. With a gradual increase in heating time, at 10800s, the temperature at observation point CH02 is 64.5℃. The temperature at the observation point noticeably increases, indicating a positive correlation. As heating time increases, the temperature at the observation point also rises.

#### Effect of internal heat source on temperature distribution

In order to analyze the influence of heat source intensity on the observation points at different distances from the heat source under the conditions of the same heat source intensity and thermal conductivity, the main calculation parameters conditions are chosen as shown in Table 5.

**Table 5:** Calculation conditions of influence of internal heat source on temperature distribution.

Number	Heat source intensity	Heat generation time	Thermal conductivity	Location Coordinates
	W/m^2^	s	W/(m^2^⋅°C)	m
1	800	10800	1.8	CH01
2	800	10800	1.8	CH02
3	800	10800	1.8	CH03
4	800	10800	1.8	CH04
5	800	10800	1.8	CH05

**Fig 6:**
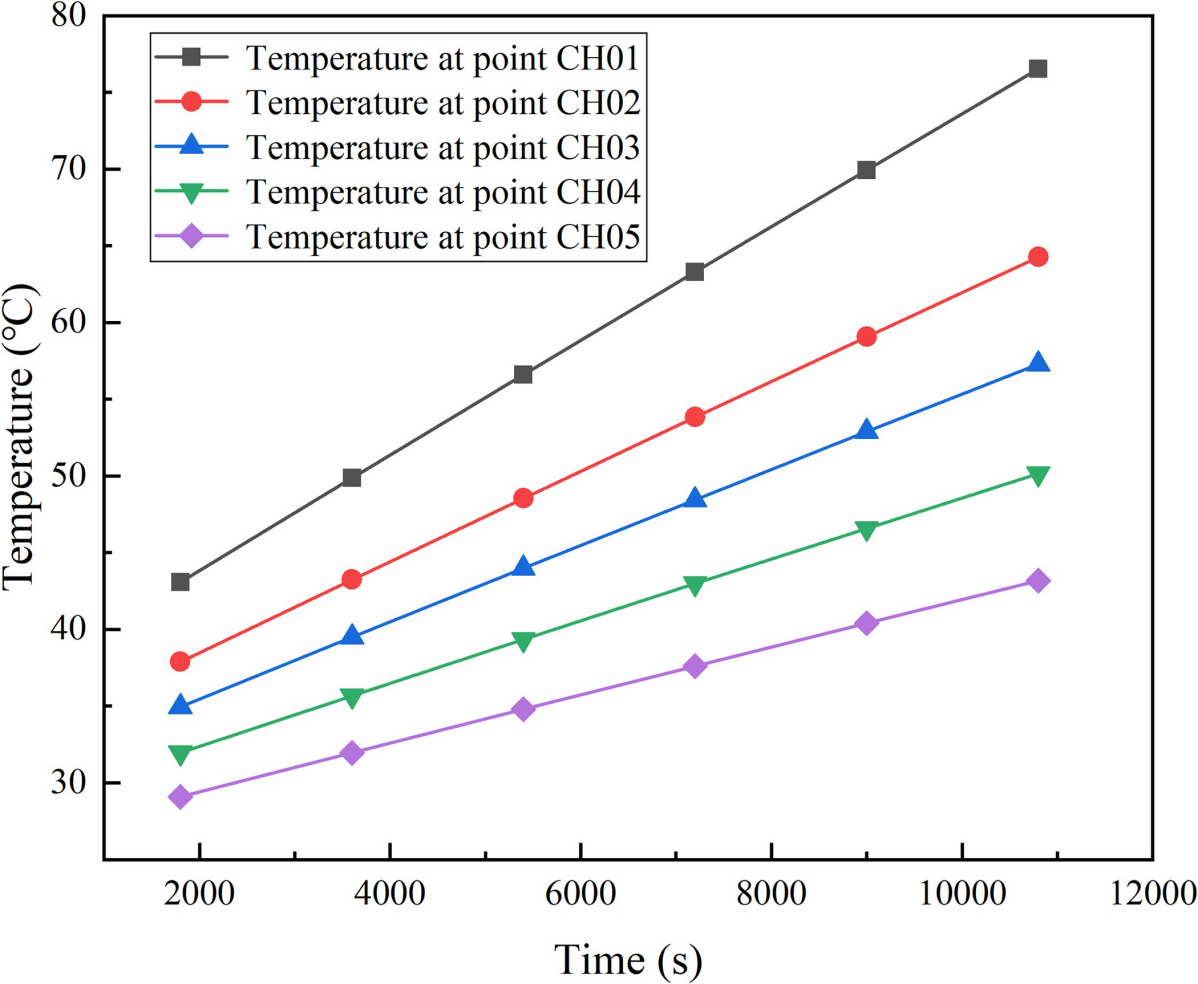
Influence of internal heat source on temperature distribution.

The temperature changes of different observation points after 3h were calculated as shown in Fig 6. It can be found that the temperature of all measurement points increases with time, but the rate of increase is not the same: comparing different observation points, the closer the location of the heat source, the higher the temperature and the rate of increase of temperature; comparing the observation points of CH01 and CH02, although the location of the heat source is the same for both points, the temperature value of observation point CH01 is greater than that of The temperature value of CH02.

## Case and discussion

**Fig 7:**
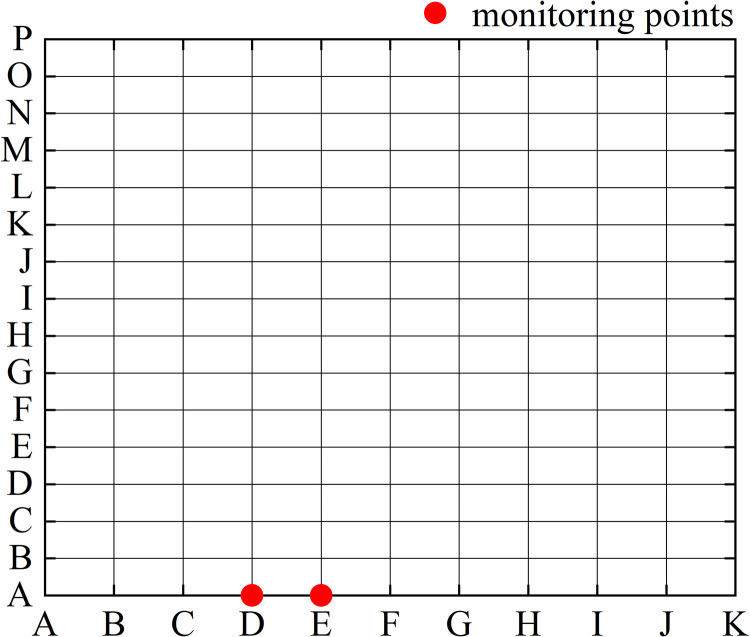
Divided grids of the goaf model.

We take a simple goaf model 10m × 15m as a case. The paraments are listed in Table 6. The simple goaf area is divided into the grids 10× 15 in Fig 7.

**Table 6:** Paraments of a simple goaf.

Number	Paraments	Values
1	*ρ*	1200 kg/m^3^
2	*c*	830 kJ/(kg·°C)
3	*λ*	1.8 W/(m·°C)
4	*α*	1.84 m^2^/s
5	*q*	W/m^3^
6	*H*_1_	2.66 W/(m^2^°C)
7	*H*_2_	2.17 W/(m^2^°C)
8	*T*_0_	283 K
9	*q*_*b*_	800 W/m^2^

*DA*(3,0) and *EA*(4,0) are selected for monitoring points. At *t* = 3600*s*, the temperature is assumed for 13.2℃ at *DA* and 12.6℃ at *EA* when the point (3,3) is a fire source. We can loop each coordinate point as a fire source, take the parameters into the points and calculate other coordinates’ temperature by Eq (7) at 3600s. The deviation value *E* at other coordinates is calculated by Eq (8). When the deviation values *E* at the monitoring points are the smallest, the coordinate point can be seen as a real fire source. Tables 7 and 8 show the temperature values at *DA* and *EA*. Table 9 shows the deviation value of *E* under different heat source points. At (1,4) and (3,3), the smallest deviation values of *E* both are 0.09. So we could see the points (1,4) and (3,3) as the fire sources. If a more accurate result is expected, more monitoring points can be used.

**Table 7:** Temperature values of *DA* at different heat source points.

coordinates	0	1	2	3	4	5	6	7	8	9
0	14.46	14.39	14.17	13.82	13.32	12.69	11.94	11.06	10.08	8.99
1	14.38	14.31	14.10	13.74	13.25	12.62	11.87	11.00	10.02	8.94
2	14.15	14.08	13.86	13.52	13.03	12.42	11.68	10.82	9.86	8.79
3	13.76	13.69	13.48	13.14	12.67	12.07	11.35	10.52	9.58	8.55
4	13.22	13.15	12.95	12.63	12.17	11.60	10.91	10.11	9.21	8.22
5	12.53	12.47	12.28	11.97	11.54	11.00	10.34	9.58	8.73	7.79
6	11.71	11.65	11.48	11.19	10.78	10.28	9.66	8.96	8.16	7.28
7	10.76	10.71	10.54	10.28	9.91	9.44	8.88	8.23	7.50	6.69
8	9.69	9.64	9.50	9.26	8.93	8.51	8.00	7.41	6.75	6.02
9	8.52	8.48	8.35	8.14	7.85	7.48	7.03	6.52	5.93	5.30
10	7.25	7.22	7.11	6.93	6.68	6.36	5.99	5.55	5.05	4.51
11	5.91	5.88	5.79	5.64	5.44	5.18	4.88	4.52	4.12	3.67
12	4.50	4.47	4.41	4.30	4.14	3.95	3.71	3.44	3.13	2.80
13	3.04	3.02	2.98	2.90	2.80	2.67	2.51	2.32	2.12	1.89
14	1.55	1.54	1.52	1.48	1.42	1.36	1.28	1.18	1.08	0.96

**Table 8:** Temperature values of *EA* at different heat source points.

coordinates	0	1	2	3	4	5	6	7	8	9
0	13.94	13.87	13.67	13.32	12.84	12.24	11.51	10.66	9.71	8.67
1	13.87	13.80	13.59	13.25	12.77	12.17	11.44	10.61	9.66	8.62
2	13.64	13.57	13.37	13.03	12.56	11.97	11.26	10.43	9.50	8.48
3	13.26	13.20	13.00	12.67	12.22	11.64	10.95	10.14	9.24	8.24
4	12.74	12.68	12.49	12.17	11.74	11.18	10.52	9.75	8.88	7.92
5	12.08	12.02	11.84	11.54	11.13	10.60	9.97	9.24	8.42	7.51
6	11.29	11.23	11.06	10.78	10.40	9.91	9.32	8.63	7.86	7.02
7	10.37	10.32	10.17	9.91	9.55	9.10	8.56	7.93	7.23	6.45
8	9.34	9.30	9.16	8.93	8.61	8.20	7.71	7.15	6.51	5.81
9	8.21	8.17	8.05	7.85	7.56	7.21	6.78	6.28	5.72	5.11
10	6.99	6.96	6.85	6.68	6.44	6.14	5.77	5.35	4.87	4.35
11	5.69	5.67	5.58	5.44	5.25	5.00	4.70	4.36	3.97	3.54
12	4.34	4.31	4.25	4.14	3.99	3.80	3.58	3.32	3.02	2.70
13	2.93	2.91	2.87	2.80	2.70	2.57	2.42	2.24	2.04	1.82
14	1.49	1.48	1.46	1.42	1.37	1.31	1.23	1.14	1.04	0.93

**Table 9:** Deviation value of *E* under different heat source points.

coordinates	0	1	2	3	4	5	6	7	8	9
0	1.84	1.74	1.44	0.95	0.27	0.63	1.67	2.88	4.25	5.76
1	1.73	1.63	1.34	0.84	0.18	0.72	1.76	2.97	4.33	5.83
2	1.41	1.31	1.02	0.53	0.17	1.01	2.03	3.22	4.56	6.03
3	0.87	0.77	0.49	0.09	0.65	1.48	2.48	3.63	4.94	6.37
4	0.14	0.09	0.27	0.72	1.34	2.14	3.10	4.21	5.46	6.84
5	0.85	0.93	1.19	1.62	2.22	2.97	3.88	4.94	6.12	7.43
6	1.99	2.07	2.31	2.71	3.27	3.98	4.83	5.81	6.92	8.14
7	3.30	3.38	3.60	3.97	4.48	5.13	5.91	6.82	7.84	8.96
8	4.79	4.85	5.05	5.39	5.85	6.43	7.14	7.95	8.87	9.88
9	6.42	6.48	6.65	6.94	7.35	7.86	8.48	9.20	10.00	10.89
10	8.17	8.22	8.38	8.62	8.97	9.41	9.93	10.54	11.23	11.99
11	10.04	10.08	10.21	10.41	10.69	11.05	11.48	11.97	12.53	13.15
12	12.00	12.03	12.13	12.28	12.49	12.77	13.09	13.47	13.90	14.37
13	14.03	14.05	14.11	14.22	14.36	14.54	14.77	15.02	15.31	15.63
14	16.10	16.11	16.14	16.20	16.27	16.36	16.48	16.61	16.75	16.91

## Conclusions

To address the challenge of locating the fire source in the air-mining zone, this study introduces a novel theoretical inversion method to analyze the heat transfer process within the air-mining zone, simplifying it into a two-dimensional heat transfer process. The unsteady-state heat transfer model of the air mining zone under specific ideal working conditions is formulated. The analytical solution for the temperature of the air mining zone, considering a single-point fire source, is obtained using the Green’s function method to derive the temperature model of the internal field of the air mining zone. The study explores the impacts of fire source intensity, heat generation time, heat conductivity coefficient, and different fire source locations on the temperature field of the air mining zone. The proposed analytical solution for the temperature field in the air-mining zone is demonstrated to be relatively accurate.

At an intensity of 100 W/m^2^, the temperature at observation point CH01 is 45.84℃. When the heat source intensity is increased to 1000 W/m^2^, the temperature at the same observation point rises to 81.2℃. Clearly, as the heat source intensity increases, the temperature at the monitoring point also increases proportionally.

The temperature at the measurement point exhibits a positive correlation with thermal conductivity and heating time. In other words, higher thermal conductivity results in a faster temperature increase, and longer heating times lead to higher temperatures.Different observation points exhibit higher temperatures and faster rates of warming when closer to the heat source. Similarly, for the same observation point, selecting a higher thermal conductivity results in higher temperature values and faster rates of warming.

## Supporting information

S1 Dataset This is the raw data from Fig 3. (XLSX)

S2 Dataset This is the raw data from Fig 4. (XLSX)

S3 Dataset This is the raw data from Fig 5. (XLSX)

S4 Dataset This is the raw data from Fig 6. (XLSX)
